# FGF21 alleviates endothelial mitochondrial damage and prevents BBB from disruption after intracranial hemorrhage through a mechanism involving SIRT6

**DOI:** 10.1186/s10020-023-00755-x

**Published:** 2023-12-04

**Authors:** Runfeng Wang, Jin Wang, Zhiguo Zhang, Bo Ma, Shukai Sun, Li Gao, Guodong Gao

**Affiliations:** Department of Neurosurgery, Tangdu Hospital, The Air Force Military Medical University, Xi’an, 710038 Shaanxi China

**Keywords:** FGF21, SIRT6, Endothelial cells, BBB, Mitochondria quality control, ICH

## Abstract

**Background:**

Disruption of the BBB is a harmful event after intracranial hemorrhage (ICH), and this disruption contributes to a series of secondary injuries. We hypothesized that FGF21 may have protective effects after intracranial hemorrhage (ICH) and investigated possible underlying molecular mechanisms.

**Methods:**

Blood samples of ICH patients were collected to determine the relationship between the serum level of FGF21 and the $\Delta$GCS%. Wild-type mice, SIRT6^flox/flox^ mice, endothelial-specific SIRT6-homozygous-knockout mice (eSIRT6^−/−^ mice) and cultured human brain microvascular endothelial cells (HCMECs) were used to determine the protective effects of FGF21 on the BBB.

**Results:**

We obtained original clinical evidence from patient data identifying a positive correlation between the serum level of FGF21 and $\Delta$GCS%. In mice, we found that FGF21 treatment is capable of alleviating BBB damage, mitigating brain edema, reducing lesion volume and improving neurofunction after ICH. In vitro, after oxyhemoglobin injury, we further explored the protective effects of FGF21 on endothelial cells (ECs), which are a significant component of the BBB. Mitochondria play crucial roles during various types of stress reactions. FGF21 significantly improved mitochondrial biology and function in ECs, as evidenced by alleviated mitochondrial morphology damage, reduced ROS accumulation, and restored ATP production. Moreover, we found that the crucial regulatory mitochondrial factor deacylase sirtuin 6 (SIRT6) played an irreplaceable role in the effects of FGF21. Using endothelial-specific SIRT6-knockout mice, we found that SIRT6 deficiency largely diminished these neuroprotective effects of FGF21. Then, we revealed that FGF21 might promote the expression of SIRT6 via the AMPK–Foxo3a pathway.

**Conclusions:**

We provide the first evidence that FGF21 is capable of protecting the BBB after ICH by improving SIRT6-mediated mitochondrial homeostasis.

**Supplementary Information:**

The online version contains supplementary material available at 10.1186/s10020-023-00755-x.

## Introduction

Intracranial hemorrhage (ICH) has increasingly become a major cause of death in modern society. The disruption of the brain‒blood barrier (BBB) is a vital event for the secondary damage caused by ICH. Under normal conditions, the BBB is responsible for preventing harmful materials from entering the central nervous system (CNS) and maintaining routine exchange with the peripheral circulatory system. Damage to the BBB aggravates brain swelling and permits inflammatory cell infiltration. Such events form a vicious cycle and cause secondary injury after ICH (Zlokovic 2008). As an essential component of the BBB, the neurovascular unit consists of cerebral microvascular endothelial cells (ECs), astrocytes, pericytes, neurons and an extracellular matrix (Hawkins and Davis 2005). Among these structures, ECs and their membrane proteins, such as tight junction proteins (TJ-ps) and adherens junction proteins (AJ-ps), execute a series of important functions (Bbb et al. 2020; Profaci et al. 2020). Due to detrimental stresses caused by ICH, the energy supply for ECs is gradually exhausted. Thus, under conditions of ICH injury, maintaining the metabolic homeostasis of ECs by preserving normal mitochondrial function could be a rational treatment strategy (Ahluwalia et al. 2021; Watts et al. 2013; Wang et al. 2018a; Chen et al. 2021).

Fibroblast growth factor 21 (FGF21), which is an endocrine molecule, belongs to the fibroblast growth factor (FGF) family. According to the literature, FGFs widely take part in maintaining the metabolic homeostasis of different cells and exerting beneficial effects in a variety of diseases (Dolegowska et al. 2019; Fisher and Maratos-Flier 2016; Tezze et al. 2019). Mouse FGF21 and human FGF21 are highly homologous (~ 75% identity) (Murata et al. 2011). Due to its unique characteristic of a low binding affinity for heparin, FGF21 is capable of crossing the BBB and binding to fibroblast growth factor receptor 1 (FGFR1) to accomplish different biological functions. According to accumulating evidence, administration of FGF21 to rodents alleviates neural damage in multiple diseases. For example, it protects endothelial cells in ischemia/reperfusion (I/R) or traumatic brain injury (Jiang et al. 2020; Chen et al. 2018), alleviates hippocampal damage in obesity-induced cognitive dysfunction (Wang et al. 2018b), prevents neuronal ferroptosis after spinal cord injury and attenuates neuronal inflammation and oxidant stress in aging and diabetes (Xu et al. 2023; Kang et al. 2020). However, few studies have evaluated the protective effects of FGF21 against ICH.

Maintaining the normal function of mitochondria is beneficial for the survival of ECs when they confront the challenges caused by ICH, and EC survival can partly preserve the integrity of the BBB. FGF21 has been proven to preserve normal mitochondrial function (Li et al. 2019), but the specific mechanism of this preservation has yet to be fully clarified. Recent studies have indicated that sirtuin 6 (SIRT6) is a regulatory factor that is vital for mitochondrial quality control. For example, SIRT6 has the ability to preserve the normal morphology of mitochondria in cardiomyocytes through PGC-1$\alpha$-AKT signaling (Yu et al. 2021). Through coactivation with Nrf2, SIRT6 exerts protective effects on mitochondria in mesenchymal stem cells (Kasai et al. 2020). Notably, endothelial SIRT6 has been demonstrated to be essential for restricting ischemic stroke size and neurological deficits by preserving BBB integrity (Liberale et al. 2020). In a preliminary experiment, we found that FGF21 fosters the transcription of SIRT6 after ICH. Hence, it is rational to explore whether FGF21 exerts protective effects after ICH in a SIRT6-dependent manner.

Therefore, in this study, we aimed to explore whether FGF21 can alleviate BBB damage and promote brain recovery after ICH. First, the clinical data of 77 ICH patients were collected and analyzed to evaluate the correlation between the serum level of FGF21 and $\Delta$GCS%. Next, given the energy-exhausted states caused by ICH and the vital role of mitochondria in preserving metabolic homeostasis, we hypothesized that FGF21 may maintain the stability of ECs and the BBB, at least partly, by alleviating mitochondrial damage.

## Materials and methods

### Patients

The Ethics Committee of the Second Affiliated Hospital of the Air Force Medical University of China approved this study (the registered number for this retrospective study is 202101–08). From September 2021 to March 2022, 77 emergency room patients aged 56.7 $\pm$ 10.9 years who suffered ICH with hematomas of 15.13 $\pm$ 2.7 ml were enrolled in this study. Patients with severe neurological or hepatic diseases or other severe systemic diseases were excluded. The Glasgow Coma Scale (GCS) was used to evaluate the severity of coma (Teasdale and Jennett 1974). The serum level of FGF21 at 24 h from the onset of ICH was measured. Patients’ GCS scores on hospital admission and at discharge were also recorded. $\Delta$GCS% indicates the following ratio: (GCS^discharge^ − GCS^admission^)/GCS^admission^. Positive values indicate neurological improvement, while negative values indicate deterioration during hospitalization. According to the $\Delta$GCS%, patients were assigned to two groups: the $\Delta$GCS% > 0 group and the $\Delta$GCS% < 0 group. The relationship between the serum level of FGF21 and the $\Delta$GCS% was determined. The detailed experimental protocols are listed in Additional file 1.

### Animals

A committee of the Second Affiliated Hospital of the Air Force Medical University of China reviewed and approved the protocols for these experiments, and these experiments were performed strictly according to the guidelines of the National Institutes of Health Guide for the Care and Use of Laboratory Animals. C57BL/6 mice (25–30 g) were purchased from the Animal Center of the Air Force Medical University. They were divided into 4 groups to determine the protective effects of FGF21 on the neural system and BBB in vivo (the detailed experimental protocols are provided in Additional file 1). SIRT6^flox/flox^ homozygous mice were purchased from the Shanghai Model Organisms Center (NM-CKO-200241). Through the crossbreeding of SIRT6^flox/flox^ mice with mice expressing Cre recombinase under the control of the vascular endothelial-specific cadherin promoter (Cdh5-CreERT2,NM-KI-200173, ShangHai model organisms), we generated Cdh5-CreERT2-SIR6^flox/flox^ mice. Next, according to the manufacturer’s instructions (T5648, Sigma‒Aldrich), we dissolved tamoxifen in corn oil and then made a mixture of tamoxifen and corn oil at a concentration of 20 mg/ml. This mixture was injected intraperitoneally (20 mg/kg) every 24 h for 5 consecutive days to obtain eSIRT6^−/−^ mice. Meanwhile, SIRT6^flox/flox^ homozygous littermates that did not receive tamoxifen injection were utilized as controls to assess the involvement of the SIRT6 pathway (the detailed experimental protocols are provided in Additional file 1). Recombinant FGF21 (C600252) was purchased from Sangon Biotech and intraperitoneally administered (1.5 mg/kg) immediately after the induction of ICH for 7 days. All mice were kept in special facilities with proper humidity and temperature and were provided with abundant food and water.

### Induction of ICH in vivo

The mice were anesthetized with 1% pentobarbital (50 ml/kg) and fixed to a stereotactic frame. An approximately 0.5 cm longitudinal incision was made along the midline of the head to expose the bregma. The coordinates of the drill hole relative to bregma were as follows: 2.0 mm lateral and 0.4 mm anterior. A microsyringe was used to vertically penetrate 3 mm into the cortex, and approximately 0.5 µl of 0.05% collagenase (C5138, Sigma) was injected into the striatum. The microsyringe was withdrawn after 6 min, and the wound was sealed. The mice in the sham group experienced all operative processes except for the injection of 0.5 µl 0.05% collagenase, which was the key difference with the ICH, ICH + Vehicle and ICH + FGF21 groups. For the ICH + Vehicle group, normal saline was employed to simulate the process of intraperitoneal injection of FGF21 in the ICH + FGF21 group. Areas of interest were selected from perihematoma spots 1.5 mm away from the hematoma (Fang et al. 2019).

### Behavioral assessments

The modified Neurological Severity Score (MNSS) was utilized to assess sensorimotor deficits in mice before and after the induction of ICH. The MNSS is capable of evaluating sensory and motor deficits through an elaborate grading system (score of 0–18) (Shi et al. 2021; Liu et al. 2023). A higher score indicates more severe damage. The tests were performed by two blinded observers. The detailed rating scale is listed in Additional file 3.

A 30° device was employed to perform the corner turn test (Shi et al. 2021). Mice were allowed to proceed toward the device and freely choose the direction of turning at the corner before and after ICH. The number of right-turn choices was recorded during 10 repeat tests by 2 blinded observers.

For the wire hanging test (Shi et al. 2021; Zhu et al. 2014), the hind limbs of each mouse were tied, and the mouse was placed on a metallic wire stretched between two posts approximately 40 cm above the ground. A soft pillow was placed under the wire to avoid injury caused by a fall. Before and after ICH, the mice were allowed to grasp the wire tightly, and the duration before falling was recorded.

### Measurement of brain water content

Seventy-two hours after ICH, the brains of the mice were removed and weighed. The data were recorded as the wet weight. Next, we put the brains into an oven for 72 h at temperatures from 95 to 100 °C. These brains were then weighed again, and the data were recorded as the dry weight. Brain water content was assessed and calculated using the following formula: (wet weight-dry weight)/wet weight × 100%.

### Magnetic resonance imaging (MRI)

After anesthetization, the mice were fixed to a special device to fix their head and restrict movement. With a Discovery MR750 3.0T scanner (General Electric Company, USA), the lesion volume of each mouse was measured 72 h after onset. T2-weighted pictures were selected. Fiji software was employed to assess and analyze the results.

### Evans blue staining

Mice were fixed in a special device with their tails anchored at an illuminated groove. After massaging the tail and temporarily blocking the blood flow, the tail vein could be easily spotted. Two hundred microliters of 2% Evans blue was injected with a microsyringe. Seventy-two hours later, the mice were sacrificed, and their brains were sliced for the following procedures. Evans blue extravasation was measured and analyzed according to a previous study (Radu and Chernoff 2013).

### Transmission electron microscopy

Mice were sacrificed and perfused with 0.9% saline and 4% paraformaldehyde at 72 h after ICH. The brains were removed and coronally sliced into 2 mm slices. Then, slices that contained a hematoma were selected and immersed in 4% glutaraldehyde overnight. We used 1% osmium tetroxide to fix these slices for 1 h. Next, the slices were dehydrated with ethanol and embedded in resin. Finally, the slices were cut into 80-nm sections and observed with a JEM-1400 electron microscope (JEOL, Tokyo, Japan).

### Cultured human brain microvascular endothelial cells (HCMECs)

Human brain microvascular endothelial cells (HCMECs) were purchased from iCell Corporation (iCell-h070, China) and cultivated in EBM-2 supplemented with penicillin‒streptomycin in cell culture plates. SIRT6 silencing was performed with a small interfering RNA targeting SIRT6 (siSIRT6) in vitro. Invalid small interfering RNA was used as a negative control (si-NC). Lipofectamine™ 2000 was employed for siRNA transfection according to the manufacturer’s instructions (18324012-012, Invitrogen, USA) after the cells reached a proper density. HCMECs were randomly pretreated with FGF21/vehicle/siSIRT6/si-NC before exposure to oxyhemoglobin. (the detailed experimental protocols are described in Additional file 1, siSIRT6: 5ʹ-CAAGUGUAAGACGCAGUACGUTT-3ʹ forward and 5ʹ-ACGUACUGCGUCUUACACUUGTT-3ʹ reverse, si-NC: 5ʹ-UUCUCCGAACGUGUCACGUTT-3ʹ forward and 5ʹ-ACG UGACACGUUCGGAGAATT-3ʹ reverse). After incubation with oxyhemoglobin for 2 h, the cell samples were collected for subsequent procedures.

### Cell viability test

Cell viability was measured using a CCK-8 kit (96992, Sigma). Approximately 10 µl of HCMECs per well were seeded in a 96-well plate according to the manufacturer’s instructions. After 24 h, the cells were assigned to different groups to receive different treatments according to the experimental protocols. Then, approximately 10 µl of CCK-8 solution was added to each well. Four hours later, a microplate reader was used to measure the absorbance at a wavelength of 450 nm.

### Permeability of endothelial cells

HCMECs were seeded into 24-well transwell chambers (1 × 10^5^ per well) with 200 µl of EBM-2 for 48 h (Adil and Somanath 2021). When cells reached a compact density, FGF21/vehicle/siSIRT6/si-NC was added to the chambers. Then, oxyhemoglobin was added to the related chambers for 2 h. After that, the medium was replaced with medium containing 1% FITC-dextran (10 mg/ml, Sigma). Three hours later, the medium was withdrawn, and the fluorescence of each well was measured at 485 nm excitation and 520 nm emission wavelengths with an EnSpire multimode microplate reader with EnSpire Manager software (PerkinElmer Company, USA). A higher fluorescence signal indicated more damage to the integrity of HCMEC monolayers.

### ELISA

Patient whole-blood samples were collected in EDTA-treated tubes and centrifuged at 12,000×*g* for 15 min to obtain the plasma. Then, with a specific ELISA kit (NeoBioscience, China), the level of FGF21 was measured. All procedures were strictly performed according to the manufacturer’s instructions.

### Western blotting

Samples of proteins were separated by SDS‒PAGE and transferred to PVDF membranes. The membranes were then blocked with 5% nonfat milk for 1 h. Next, specific primary antibodies were added to the box containing the blocked membranes, and the membranes were incubated overnight. After rinsing twice, the membranes were incubated with the appropriate corresponding horseradish peroxidase–conjugated anti-rabbit secondary antibodies (1:5000, AS014, ABclonal) for 2 h. Then, pictures of targeted proteins were captured with the assistance of a Bio-Rad Imaging System (Bio-Rad). ImageJ was employed to analyze the bands. The primary antibodies utilized in this experiment included anti-ZO-1 (1:1000, 7773-1-AP, Proteintech), anti-occludin (1:1000, 272601-1-AP, Proteintech), anti-SIRT6 (1:1000, 13572-1-AP, Proteintech), anti-mfn1 (1:1000, 14739, Cell Signaling), anti-mfn2 (1:1000, 11925, Cell Signaling), and anti-Drp1 (1:1000, 14647, Cell Signaling).

### Immunofluorescence (IF) and terminal deoxynucleotidyl transferase-mediated dUTP nick end labeling (TUNEL) staining

After dehydration, the mouse brains were cut into 30 µm slices and incubated with 1% Triton X-100. Then, the slices were incubated with primary antibodies such as anti-CD31 (1:500, AF806, R&D) and anti-SIRT6 (1:500, 13572-1-AP, Proteintech) overnight at 4 °C. The slices were incubated with species-specific secondary antibodies with different fluorescence signals for 2 h. The nuclei were labeled with DAPI (1:1000, Invitrogen). All slices were imaged with an A1 Si confocal microscope (Nikon) and analyzed by Fiji software. TUNEL staining was performed strictly according to the manufacturer’s instructions (C1086, Beyotime). Areas of interest were selected from perihematoma spots 1.5 mm away from the hematoma (Fang et al. 2019).

Cell samples were seeded on confocal plates. After cultivation to an appropriate density, paraformaldehyde was utilized to fix the samples. Then, the same protocols described above were utilized to treat the cell samples.

### Measurement of mitochondrial morphology and function

HCMECs were cultured on a confocal plate. After the cells reached the appropriate density and were treated with different protocols, approximately 10 nM MitoTracker Red (M22426, Invitrogen) was added to the plate, and the cells were incubated for 30 min. Thereafter, with a fluorescence microscope (A1 Si, Nikon), the morphology of mitochondria was captured and then analyzed with Fiji software according to a previous study (Valente et al. 2017). Additionally, 10 nM MitoSOX Red (M36008, Thermo) was added to the plate to measure the levels of ROS in mitochondria, and the results were analyzed with Fiji software. In addition, HCMECs were incubated with 10 nM JC-1 (C2006, Beyotime) for 30 min to assess the depolarization of the membrane. Commercial assay kits were used to measure manganese superoxide dismutase (MnSOD) activity (JL20470, Jiang Lai). For ATP measurement, according to the manufacturer’s instructions (BC0300, Solarbio), samples were lysed and centrifuged. The supernatants were mixed with the ATP detection working solution, and the ATP levels of the different groups were measured with a standard curve.

### Reverse transcription and quantitative real-time PCR

Real-time PCR was applied to assess gene expression in samples. Total RNA was extracted from HCMEC samples using TRI Reagent (Invitrogen Carlsbad, CA) according to the manufacturer’s protocols. Then, the cellular RNA was converted to cDNA in a final volume of 20 μl. All RT‒PCR experiments were performed using Master Mix provided by Thermo Fisher. Each reaction (20 μl) contained 2 μl cDNA, 400 fmol of each primer and 10 μl of Master Mix. The following primers were employed: for SIRT6, 5ʹ-GCT TCC TGG TCA GCC AGA-3ʹ (forward) and 5ʹ-CTT GGC ACA TTC TTC CAC AA-3ʹ (reverse); for β-actin, 5ʹ-GCA CAG AGC CTC GCC TT-3ʹ (forward) and 5ʹ-GTT GTC GAC GAC GAG CG-3ʹ (reverse). All qPCR assays were performed in triplicate in a 96-well plate according to the manufacturer’s protocol. The results were normalized against those of β-actin and expressed as fold changes for the relative mRNA expression levels.

### Statistical analysis

SPSS Statistics 20.0 (IBM) was employed to analyze all data. The mean and standard deviation are used for normally distributed data. The median is used for nonnormally distributed data. Categorical data are presented as frequencies. Univariate and multivariate logistic regression models were used to explore independent risk factors for unfavorable outcomes. Multiple groups were compared using one-way analysis of variance (ANOVA) or two-way analysis of variance (ANOVA) followed by Tukey’s multiple comparison test. Student’s t tests were used to compare 2 groups. Spearman correlation analysis was used to test the correlation between two quantitative variables. GraphPad Prism 6.0 software was employed for statistical analysis and drawing. Fiji software was used to quantify the targeted areas and fluorescence signals. Differences with P values < 0.05 were defined as statistically significant.

## Results

### Serum levels of FGF21 are positively correlated with GCS scores

**Fig. 1:**
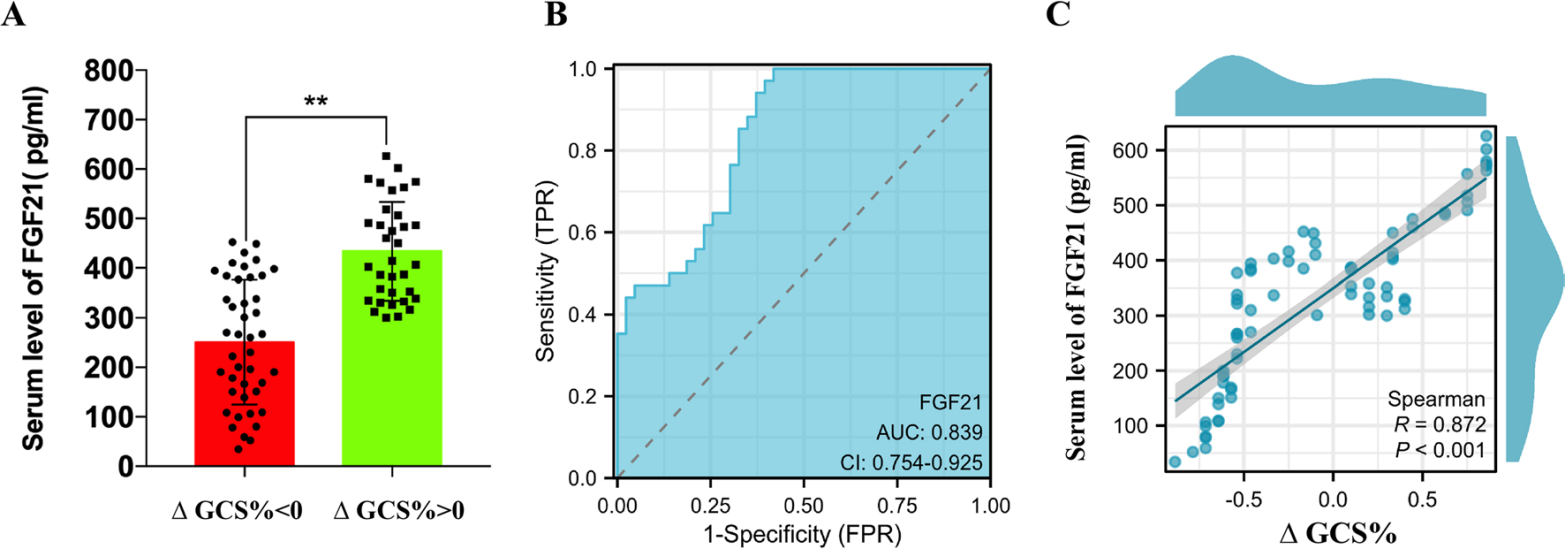
Serum levels of FGF21 are positively correlated with the $\Delta$GCS%. **A** There was statistically significant upon serum levels of FGF21 between $\Delta$GCS% < 0 group and $\Delta$GCS% > 0 group. **B** ROC curve analysis was employed to assess the ability of serum FGF21 levels to discriminate patients’ $\Delta$GCS% (Area under the curve: 0.839). (C) A positive correlation was found between the serum levels of FGF21 and the $\Delta$GCS% (r = 0.872, ^***^P < 0.001). ^**^P < 0.01 by student’s t test (**A**). Values are presented as mean $\pm$ SD

A total of 77 ICH patients were enrolled in this experiment. Detailed information regarding the patients is listed in Table 1. There were no differences between the 2 subgroups regarding age, sex, BMI or hematoma volume. As Fig. 1A reveals, the serum level of FGF21 in the $\Delta$GCS% > 0 group was higher than that in the $\Delta$GCS% < 0 group after ICH (^**^P < 0.01). Furthermore, receiver operating characteristic (ROC) curve analysis was employed to assess the ability of plasma FGF21 levels to discriminate patients’ coma severity (Fig. 1B, AUC: 83.9%). In addition, a positive correlation was found between the serum level of FGF21 and the $\Delta$GCS% (Fig. 1C, r = 0.872, P < 0.001).

**Table 1:** Characteristics of the study population

	$\Delta$ GCS > 0	$\Delta$ GCS < 0	Crude OR (95%CI)	Crude P value	Adjusted OR (95% CI)	Adjusted P value
Gender						
Female	17 (50%)	20 (46.51%)	1.150 (0.467,2.830)	0.761		
Male	17 (50%)	23 (53.49%)				
Smoking						
No	21 (61.76%)	16 (37.21%)	2.726 (1.078,6.894)	0.034	0.095 (0.004,2.262)	0.146
Yes	13 (38.24%)	27 (62.79%)				
Hypertension						
No	24 (70.59%)	20 (46.51%)	2.76 (1.067,7.140)	0.036	1.875 (0.116,30.174)	0.658
Yes	10 (29.41%)	23 (53.49%)				
Dyslipidaemia						
No	18 (52.94%)	22 (51.16%)	1.074 (0.436,2.643)	0.877		
Yes	16 (47.06%)	21 (48.84%)				
Diabetes						
No	22 (64.71%)	18 (41.86%)	2.546 (1.006,6.443)	0.048	1,867 (0.044,78.763)	0.744
Yes	12 (35.29%)	25 (58.14%)				
Age	54.35 $\pm$ 9.84	58.56 $\pm$ 11.50	1.037 (0.993,1.083)	0.097		
BMI	23.76 $\pm$ 2.487	23.70 $\pm$ 2.50	0.989 (0.824,1.187)	0.906		
Hematoma volume (ml)	14.62 $\pm$ 2.57	15.53 $\pm$ 2.78	1.138 (0.958,1.351)	0.141		
FGF21 (pg/ml)	434.15 $\pm$ 99.6	250.84 $\pm$ 125.8	0.99 (0.98,0.99)	< 0.001	0.981 (0.97,0.99)	< 0.001

### FGF21 is capable of alleviating brain injury after ICH

**Fig. 2:**
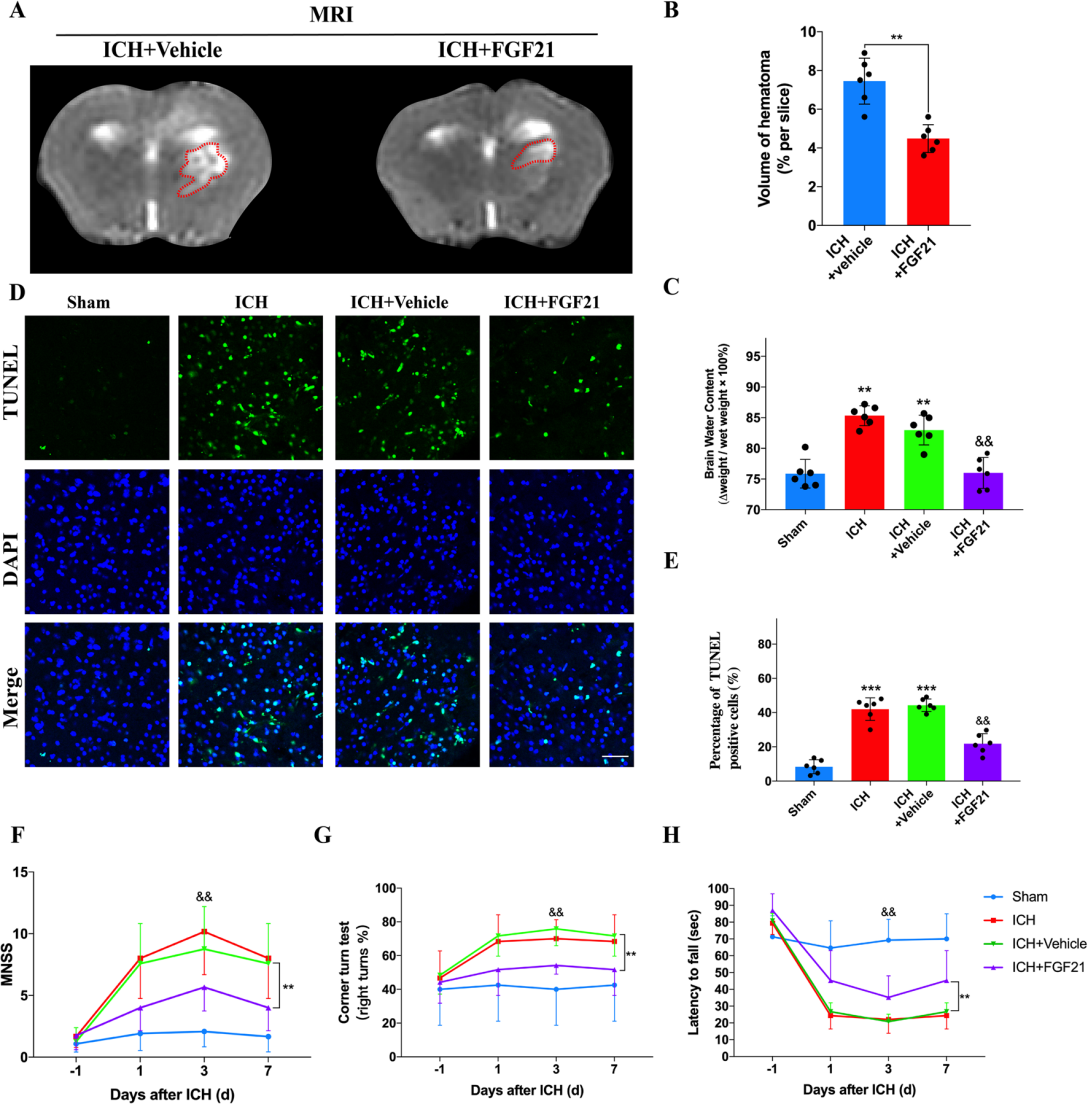
FGF21 administration is capable of reducing lesion volume, alleviating brain edema, reducing neural apoptosis and improving neurological function after ICH. **A**, **B** T2-weighted MRI images and statistics analysis 72 h after ICH. MRI red dot area: hematoma lesion. n = 6 per group. **C**–**E** Brain water content and the TUNEL stain for each group 72 h after ICH. n = 6 per group. **F**–**H** Sensorimotor tests were performed by MNSS (**F**), Corner turn test (**G**) and Latency to fall test (**H**) before and after ICH. n = 12 per group. Scale bar: 50um. ^**^P < 0.01 indicates ICH + Vehicle vs ICH + FGF21 by student’s t test (**B**), ^**^P < 0.01, ^***^P < 0.001 vs sham group and ^&&^P < 0.01 vs ICH + Vehicle group by one-way ANOVA (**C**, **E**), ^**^P < 0.01 indicates difference between two groups and ^&&^P < 0.01 indicates ICH + Vehicle vs ICH + FGF21 group within a single day by two-way ANOVA (**F**–**H**). Values are presented as mean $\pm$ SD

In mice, several methods were employed to verify the protective effects of FGF21 on the brain after ICH, such as MRI, sensorimotor tests, brain water content tests and TUNEL staining. First, according to MRI findings, the lesion volume of the ICH + FGF21 group was dramatically smaller than that of the ICH + Vehicle group at 72 h after ICH (Fig. 2A, B, ^**^P < 0.01). Next, FGF21 administration distinctly restricted the increase in the brain water content in the ICH + FGF21 group compared to the ICH + Vehicle group at 72 h after ICH. (Fig. 2C, ^&&^P < 0.01). Meanwhile, TUNEL staining demonstrated that the proportion of apoptotic cells in the ICH + FGF21 group was significantly lower than that in the ICH + Vehicle group at 72 h after ICH (Fig. 2D, E, ^&&^P < 0.01). Furthermore, as Fig. 2F–H shows, compared to vehicle treatment, FGF21 administration improved the performance of the ICH + FGF21 group in the MNSS test, wire hanging test and corner test after ICH.

### FGF21 protects the BBB after ICH

**Fig. 3:**
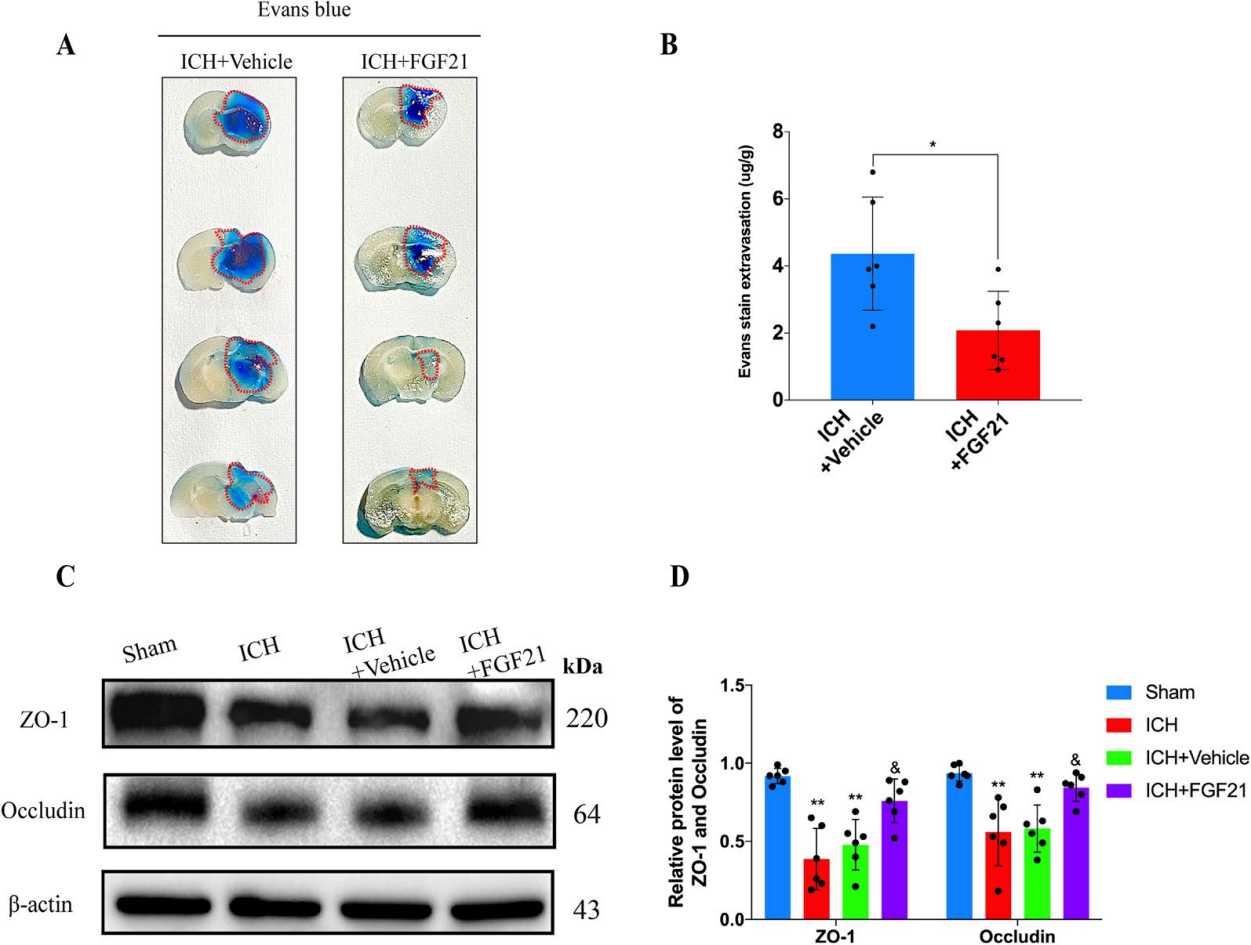
FGF21 administration protects the BBB in vivo after ICH. **A**, **B** Representative images and statistics analysis of Evans blue stain for each group 72 h after ICH. Red dot area: Evans blue volume. n = 6 per group. **C**, **D** Western blots and statistics analysis for ZO-1 and Occludin 72 h after ICH in vivo. n = 6 per group. ^*^P < 0.05 indicates ICH + Vehicle vs ICH + FGF21 by student’s t test (**B**), ^**^P < 0.01 vs Sham group and ^&^P < 0.05 vs ICH + Vehicle group by two-way ANOVA (**D**). Values are presented as mean $\pm$ SD

After ICH onset, disruption of the BBB acts as a vital push that triggers a domino effect. Thus, we further assessed alterations in the BBB after treatment with FGF21 in vivo. Comparison of the results of Evans blue staining between the ICH + Vehicle and ICH + FGF21 groups, FGF21 administration was fully capable of reducing the amount of Evans blue extravasation in the brain 72 h after ICH (Fig. 3A, B, ^*^P < 0.05), indicating its protective effects on the BBB. In addition, ICH injury dramatically reduced the levels of ZO-1 and occludin in the ICH and ICH + Vehicle groups (Fig. 3C, D, ^**^P < 0.01). However, in the ICH + FGF21 group, FGF21 partly reversed the levels of ZO-1 and occludin (^&^P < 0.05). Both ZO-1 and occludin are tight junction proteins. These proteins are critical for maintaining the normal permeability of the BBB (Abbruscato et al. 2002; Fischer et al. 2002). Thus, in light of our results, FGF21 might have the ability to prevent BBB disruption after ICH and maintain the normal function of ECs.

### FGF21 protects endothelial cells and maintains mitochondrial dynamics in vitro

**Fig. 4:**
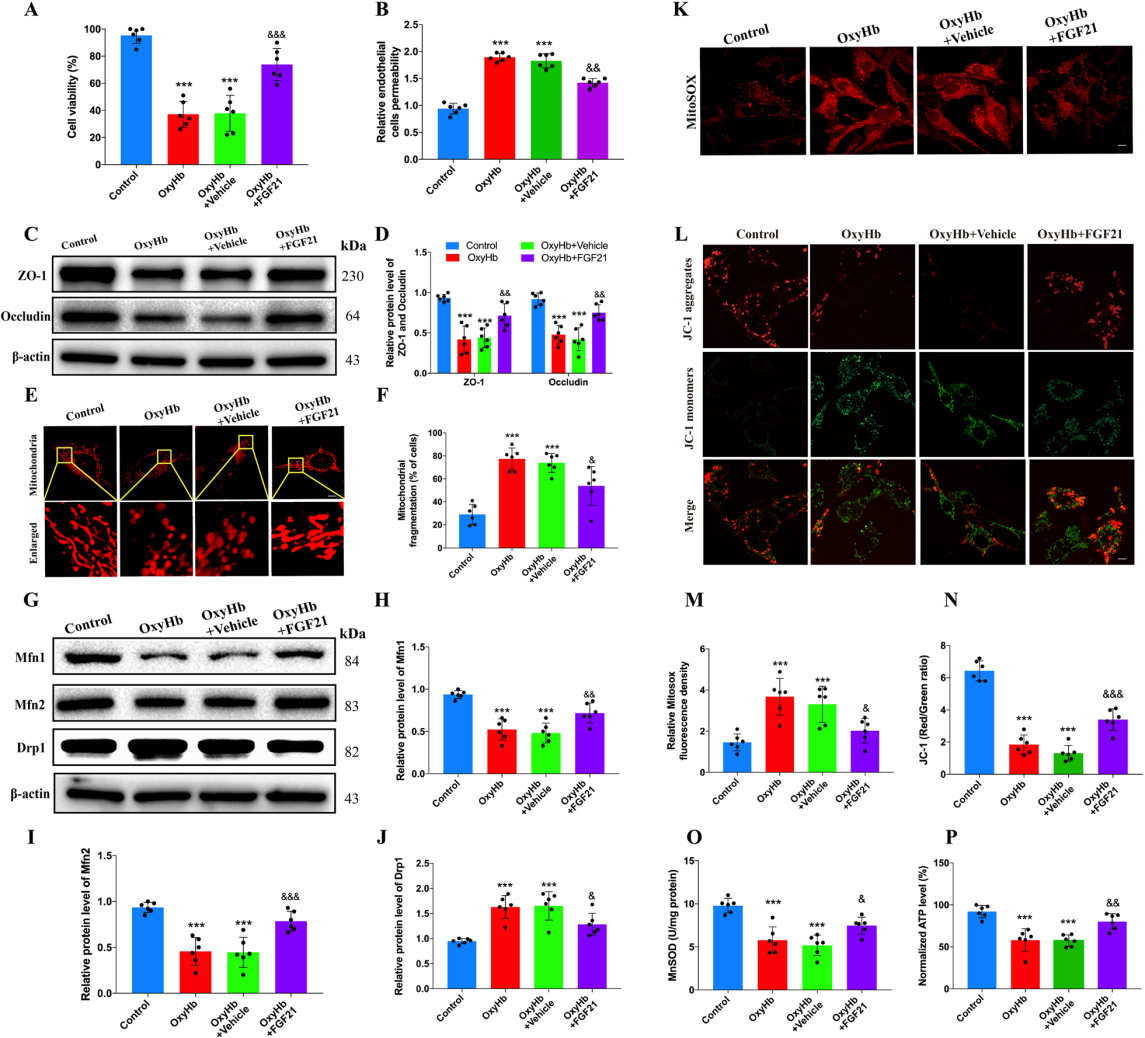
FGF21 is capable of protecting endothelial cells in vitro by maintaining normal mitochondrial morphology and function. **A**, **B** The cell viability test and endothelial cell permeability test for each group. n = 6 per group. **C**, **D** Western blots and statistics analysis for ZO-1 and occludin in vitro. n = 6 per group. **E**, **F** Representative images of Mitotracker stain and statistics analysis for each group. n = 6 per group. **G**–**J** Western blots and statistics analysis for Mfn1, Mfn2 and Drp1 in vitro. n = 6 per group. **K**, **M** Representative images of MitoSOX stain and statistics analysis for each group in vitro. n = 6 per group. **L**, **N** Representative images of JC-1 and statistics analysis for each group. n = 6 per group. **O**, **P** MnSOD and ATP test for each group. n = 6 per group. Scale bar: 10um. ^***^P < 0.001 vs Control group and ^&^P < 0.05, ^&&^P < 0.01, ^&&&^P < 0.001 vs OxyHb + Vehicle group by one-way ANOVA (**A**, **B**, **F**, **H**–**J** and **M**–**P**), ^***^P < 0.001 vs Control group and ^&&^P < 0.01 vs OxyHb + Vehicle group by two-way ANOVA (**D**). Values are presented as mean $\pm$ SD

To further demonstrate certain protective effects of FGF21 in vitro, oxyhemoglobin was utilized to mimic ICH injury (Shen et al. 2021), and HCMECs were cultivated and subjected to different treatments. As the cell viability test revealed, under conditions of damage caused by oxyhemoglobin, FGF21 treatment reduced cell death compared to that observed in the vehicle-treated group (Fig. 4A, ^&&&^P < 0.001). In addition, as Fig. 4B reveals, oxyhemoglobin impaired the integrity of the HCMEC monolayer, which led to increased permeability of the HCMEC layer compared to that of the control group (^***^P < 0.001). However, in the oxyhemoglobin + FGF21 group, the permeability of the HCMEC monolayer was partly restored compared to that of the vehicle-treated group (^&&^P < 0.01). These results indicated that FGF21 not only prevented HCMECs from dying after oxyhemoglobin injury but also partially maintained the integrity of the HCMEC monolayer. Furthermore, FGF21 treatment partially reversed the decrease in ZO-1 and occludin after oxyhemoglobin injury (Fig. 4C, D, ^&&^ P < 0.01).

Given the pivotal role of mitochondria in mediating the energy expenditure of ECs (Kluge et al. 2013; Szewczyk et al. 2015), we specifically examined alterations in mitochondrial morphology and function. As MitoTracker staining revealed, in the oxyhemoglobin + vehicle group, dramatically swollen and fragmented mitochondria were observed. The double-layer membrane structure of mitochondria lost its normal shape. Conversely, in the FGF21-treated group, the morphology of mitochondria was less fragmented and swollen than that of mitochondria in the vehicle-treated group (Fig. 4E, F, ^&^P < 0.05). We also measured the levels of Mfn1, Mfn2, and Drp1 (Bertholet et al. 2016; Bliek et al. 2013). As Fig. 4G–J reveals, the levels of Mfn1 and Mfn2 were decreased after oxyhemoglobin injury (^***^P < 0.001). However, FGF21 partially inhibited the decrease in Mfn1 and Mfn2 in the oxyhemoglobin + FGF21 group (^&&^P < 0.01, ^&&&^P < 0.001). In addition, the level of Drp1 was increased after oxyhemoglobin impairment (^***^P < 0.001). However, FGF21 inhibited the increase in Drp1 in the oxyhemoglobin + FGF21 group (^&^P < 0.05). MitoSOX staining was employed to measure the oxidative stress of mitochondria. As Fig. 4K and M reveal, the level of reactive oxygen species (ROS) was dramatically lower in the FGF21-treated group after impairment by oxyhemoglobin than in the vehicle-treated group (^&^P < 0.05). In addition, FGF21 treatment inhibited the decrease in mitochondrial membrane potential (Fig. 4L, N, ^&&&^P < 0.001). Furthermore, FGF21 was responsible for the restoration of MnSOD and ATP levels in the oxyhemoglobin + FGF21 group, which was not observed in the vehicle-treated group (Fig. 4O, P, ^&^P < 0.05, ^&&^P < 0.01). Taken together, these results indicate that FGF21 has the ability to protect HCMECs by preserving normal mitochondrial morphology and function.

### SIRT6 is involved in mediating the protective effects of FGF21

**Fig. 5:**
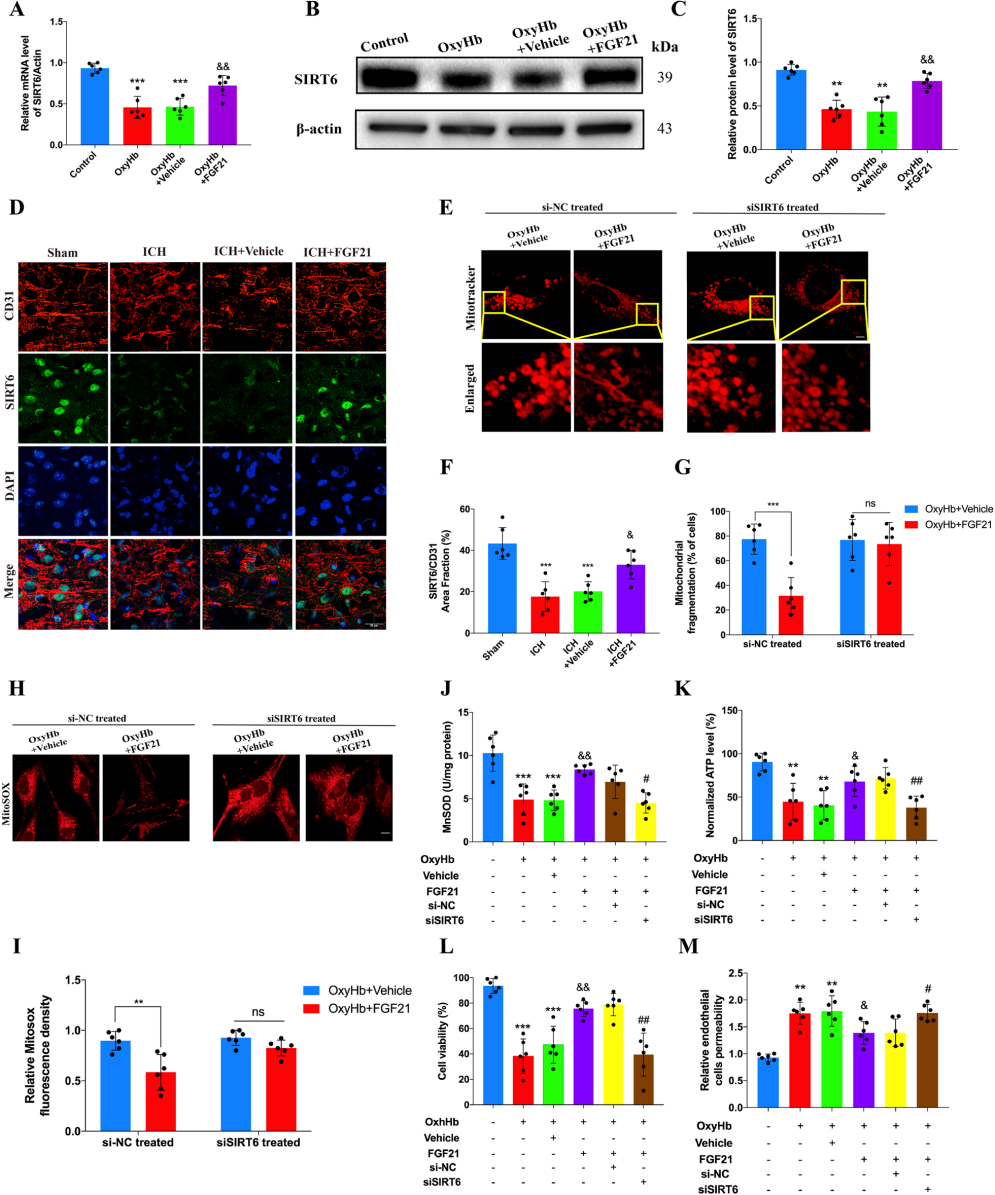
SIRT6 is involved in mediating the protective effects of FGF21 for mitochondria and ECs in vitro. **A**–**C** Western blots and qPCR for SIRT6 among different groups in vitro. n = 6 per group. **D**, **F** Representative images of immunofluorescence stain and statistics analysis for CD31 and SIRT6 among different groups 72 h after ICH in vivo. Red: CD31, Green: SIRT6, Blue: DAPI. n = 6 per group. **E**, **G** Representative images of Mitotracker stain and statistics analysis for si-NC treated group and siSIRT6 treated group. n = 6 per group. **H**, **I** Representative images of MitoSOX stain and statistics analysis for si-NC treated group and siSIRT6 treated group. n = 6 per group. **J**, **K** MnSOD and ATP test for si-NC treated and siSIRT6 treated group. n = 6 per group. **L**, **M** Cell viability and relative permeability test for si-NC treated group and siSIRT6 treated group. n = 6 per group. Scale bar: 20um (**D**) and Scale bar: 10 um (**E**, **H**). ^**^P < 0.01, ^***^P < 0.001 vs Control group and ^&&^P < 0.01 vs OxyHb + Vehicle group by one-way ANOVA (**A**, **C**), ^***^P < 0.001 vs Sham group and ^&^P < 0.05 vs ICH + Vehicle group by one-way ANOVA (**F**), ^**^P < 0.01, ^***^P < 0.001 indicates OxyHb + Vehicle vs OxyHb + FGF21 group by two-way ANOVA (**G**, **I**), ^**^P < 0.01, ^***^P < 0.001 vs Control group, ^&^P < 0.05, ^&&^P < 0.01 vs OxyHb + Vehicle group and ^#^P < 0.05, ^##^P < 0.01 vs OxyHb + FGF21 + si-NC group by one-way ANOVA (**J**–**M**), ns: no significant. Values are presented as mean $\pm$ SD

Accumulating evidence has suggested that SIRT6 plays a crucial role in mediating normal mitochondrial morphology and function, especially during certain harmful events, such as ischemic/reperfusion injury, calorie restriction, and oxidative stress (Yu et al. 2021; Fan et al. 2019; Cheng et al. 2016; Singh et al. 2018). Thus, we examined the alterations in SIRT6 mRNA and protein levels among the different groups. Intriguingly, FGF21 inhibited the decrease in SIRT6 mRNA caused by oxyhemoglobin injury (Fig. 5A, ^&&^P < 0.01). Compared to that in the vehicle-treated group, the protein level of SIRT6 was partially reversed in the FGF21-treated group (Fig. 5B, C, ^&&^P < 0.01). These results were in line with those of immunofluorescence staining obtained in vivo (Fig. 5D, F, ^&^P < 0.05).

To further clarify the underlying mechanism, SIRT6-siRNA and control siRNA (Sangon Biotech, China) were used to determine the role of SIRT6 in mediating the related protective effects on FGF21. According to MitoTracker staining, in the si-NC group, FGF21 partially preserved normal mitochondrial morphology, which was consistent with previous results (Fig. 5E, G, ^***^P < 0.001). However, in the siSIRT6 group, this beneficial effect was thoroughly diminished. Furthermore, as MitoSOX staining revealed, after transfection with siSIRT6, FGF21 failed to alleviate oxidative stress in mitochondria compared to that of the si-NC group (Fig. 5H, I, ^**^P < 0.01). Additionally, FGF21 lost the ability to reverse the levels of MnSOD and ATP after SIRT6 silencing (Fig. 5J, K, ^#^P < 0.05, ^##^P < 0.01).

For HCMECs, the cell viability and permeability of ECs in the si-NC group were still improved by FGF21 after oxyhemoglobin injury. However, in the siSIRT6 group, this protective effect vanished (Fig. 5L, M, ^#^P < 0.05, ^##^P < 0.01). Therefore, because SIRT6 silencing offset the protective effects of FGF21, we conclude that SIRT6 may be involved in the protective effects of FGF21 on HCMECs and mitochondria.

### ***FGF21 fails to protect mitochondrial morphology and function in eSIRT6***^***−/−***^*** mice after ICH***

**Fig. 6:**
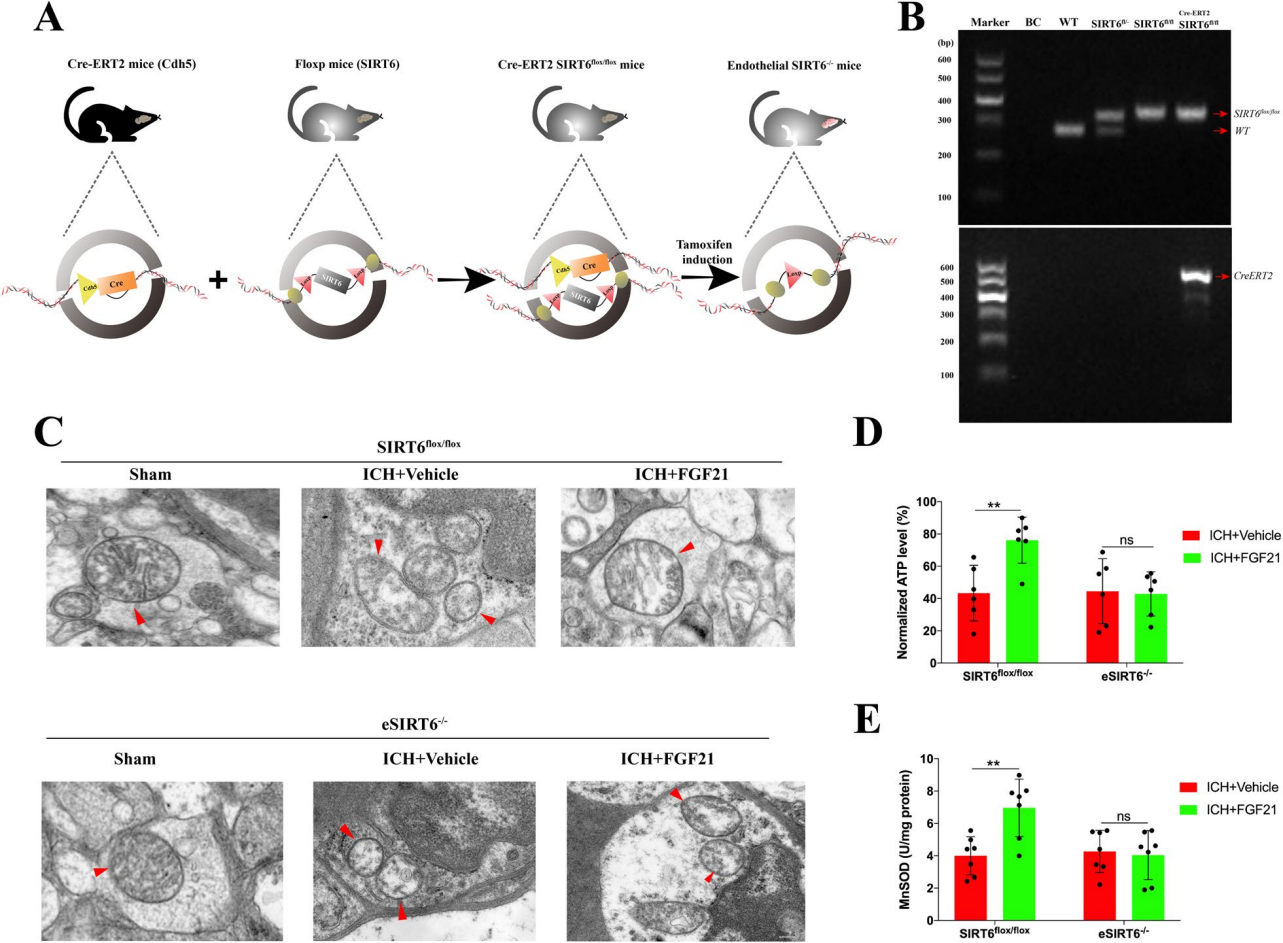
FGF21 fails to protect mitochondrial morphology and function in eSIRT6^−/−^ mice after ICH. **A** Schematic Figures for the construction of eSIRT6^−/−^ mice. **B** Genomic PCR for WT mice, SIRT6^flox/−^ mice, SIRT6^flox/flox^ mice and Cre-ERT2 SIRT6^flox/flox^ mice. **C** Representative transmission electron microscopy images for mitochondria among SIRT6^flox/flox^ mice and eSIRT6^−/−^ mice. Red triangle: representative mitochondria. **D**, **E** MnSOD and ATP test for ECs extracted from SIRT6^flox/flox^ and eSIRT6^−/−^ mice 72 h after ICH injury. n = 6 per group. Scale bar: 0.25 um. ^**^p < 0.01 indicates ICH + Vehicle vs ICH + FGF21 group by two-way ANOVA (**D**, **E**). ns: no significant. Values are presented as mean $\pm$ SD

To better demonstrate the mechanisms in vivo, eSIRT6^−/−^ mice and SIRT6^flox/flox^ mice were constructed to further reveal the cooperative relationship between FGF21 and SIRT6. During the experiments, there were no differences regarding mortality, body weight or other biological characteristics between eSIRT6^−/−^ and SIRT6^flox/flox^ littermates. The schematic shows the construction of eSIRT6^−/−^ mice (Fig. 6A). Genomic PCR was performed for WT mice, SIRT6^flox/−^ mice, SIRT6^flox/flox^ mice and Cre-ERT2 SIRT6^flox/flox^ mice (Fig. 6B). Compared to the effects observed in SIRT6^flox/flox^ littermates, according to the ultrastructure captured by transmission electron microscopy in fresh slices, the protective effects of FGF21 on mitochondrial morphology were abolished in eSIRT6^−/−^ mice (Fig. 6C). CD31 MicroBeads (130-0970418, Miltenyi Biotec, Germany) were utilized to select endothelial cells from the brains of neonatal SIRT6^flox/flox^ mice and eSIRT6^−/−^ mice. Then, the cell samples were cultivated to determine alterations in mitochondrial function among the different treatment groups. As Fig. 6D and E reveal, FGF21 failed to reverse the decrease in the levels of ATP and MnSOD in the eSIRT6^−/−^ group. Therefore, FGF21 might not fully exert protective effects on mitochondria without the presence of SIRT6.

### ***FGF21 fails to protect neurofunction and the BBB in eSIRT6***^***−/−***^*** mice after ICH***

**Fig. 7:**
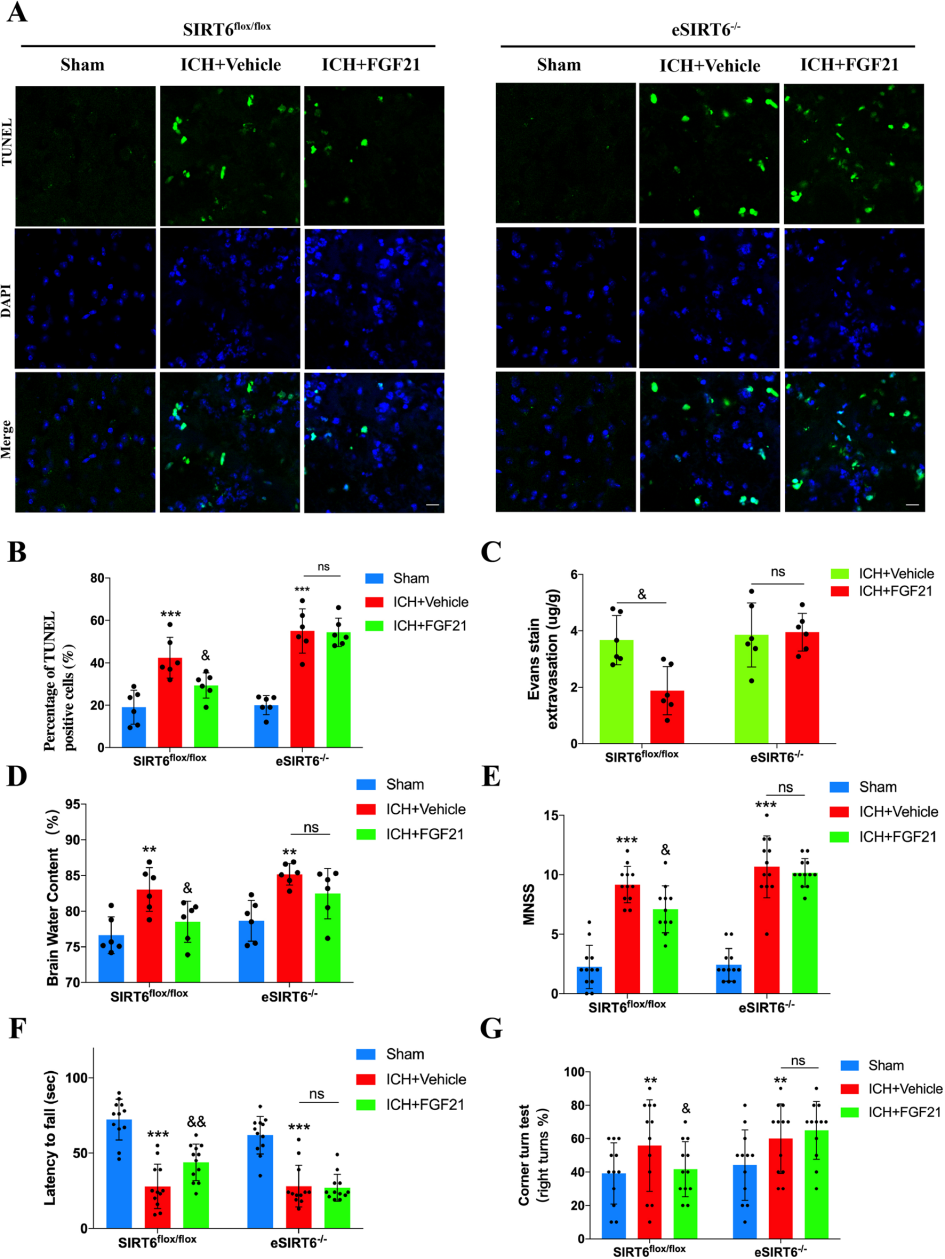
FGF21 fails to protect neurofunction and the BBB in eSIRT6^−/−^ mice after ICH. **A**, **B** Representative images of TUNEL stain for SIRT6^flox/flox^ and eSIRT6^−/−^ mice 72 h after ICH. n = 6 per group. **C**, **D** Evans stain and Brain water content test for SIRT6^flox/flox^ and eSIRT6^−/−^ mice 72 h after ICH. n = 6 per group. **E**–**G** Sensorimotor tests for SIRT6^flox/flox^ and eSIRT6^−/−^ mice 72 h after ICH. n = 12 per group. Scale bar: 50 um. ^**^P < 0.01, ^***^P < 0.001 vs Sham group and ^&^P < 0.05, ^&$^P < 0.01 vs ICH + Vehicle group by two-way ANOVA **B**–**G**. ns: no significant. Values are presented as mean $$\pm$$ SD

According to TUNEL staining, Evans blue staining and the brain water content test, FGF21 retained the ability to protect the BBB and the neural system in SIRT6^flox/flox^ mice compared to that of the vehicle-treated group 72 h after ICH (Fig. 7A–D). In addition, according to the results of multiple sensorimotor tests, FGF21 still improved the performance of ICH-injured mice in the SIRT6^flox/flox^ group, which was in line with the former results (Fig. 7E–G). Unfortunately, in the eSIRT6^−/−^ group, there was no difference between vehicle- and FGF21-treated mice based on the results of TUNEL staining, Evans blue staining, brain water content testing and sensorimotor tests. Such evidence indicated that FGF21 failed to fully exert its protective effects on neurofunction and the BBB without SIRT6 in vivo.

### FGF21 increases the expression of SIRT6 through the AMPK–FoxO3a pathway

**Fig. 8:**
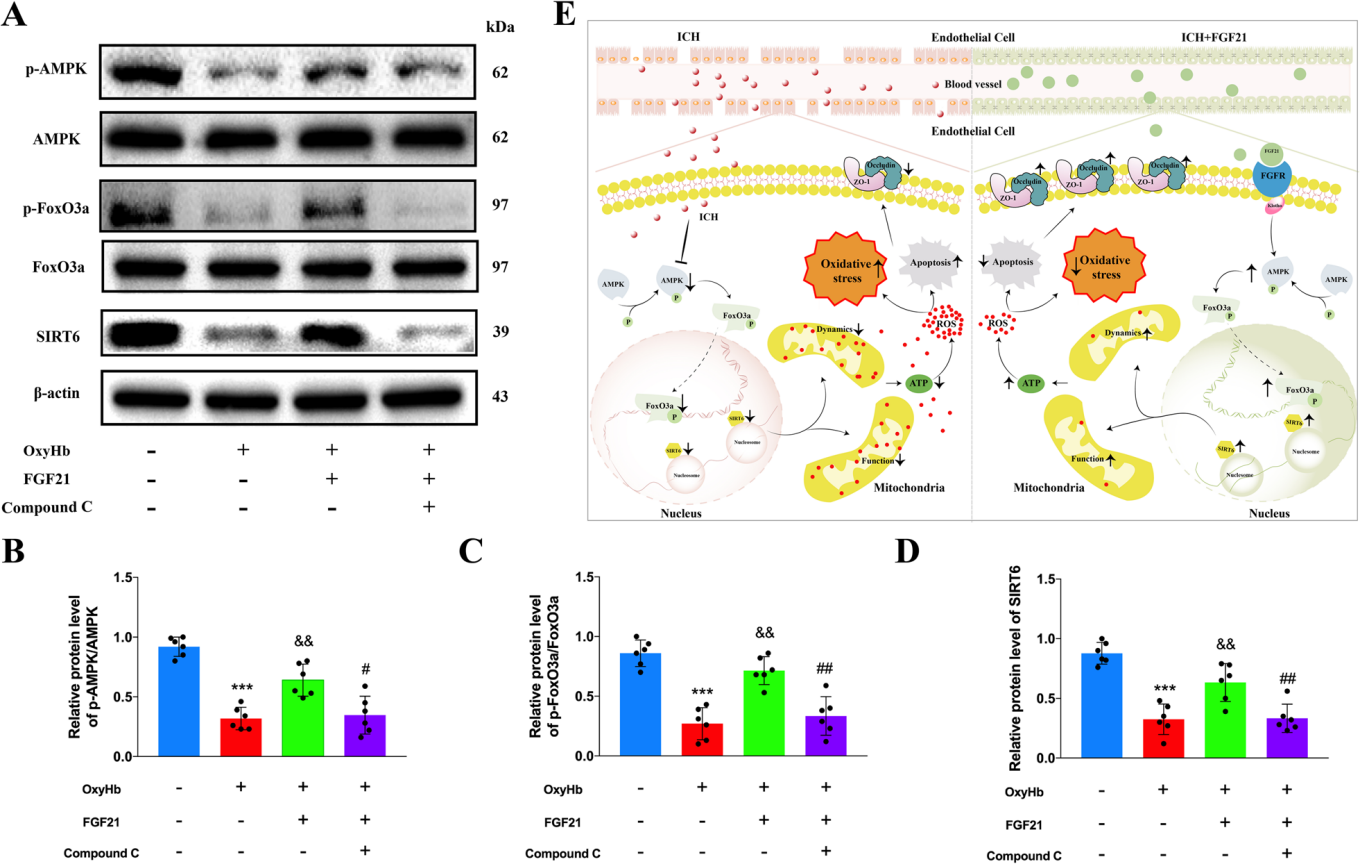
FGF21 increases the expression of SIRT6 through the AMPK–FoxO3a pathway. **A**–**D** Western blots and statistics analysis for AMPK, p-AMPK, FoxO3a, p-FoxO3a and SIRT6 among different groups. n = 6 per group. **E** Schematic figure displays the mechanism of FGF21 exerts protective effects for BBB after ICH injury. FGF21 increased the level of SIRT6 through AMPK–FoxO3a pathway, further improved the mitochondrial morphology and function of ECs. ^***^P < 0.001 vs Control group, ^&&^P < 0.01 vs OxyHb group, ^#^P < 0.05, ^##^P < 0.01 vs OxyHb + FGF21 group by one-way ANOVA (**B**–**D**). ns: no significant. Values are presented as mean $\pm$ SD

Based on these in vitro and in vivo results, FGF21 influences the expression of SIRT6. According to published literature, SIRT6 is a novel direct target of FoxO3a (Zhang et al. 2019; Dong et al. 2020). As shown in Additional file 2: Figure S2C, in the present study, SIRT6 mRNA in ECs was partly decreased after siRNA-FoxO3a interference in vitro, which is consistent with previous literature. Furthermore, AMPK–FoxO3a signaling is a potential downstream pathway for FGF21 (Zhou et al. 2019), and it is crucial for cell survival under energy stress (Fasano et al. 2019). Thus, we explored the levels of such proteins to verify whether FGF21 can influence the expression of SIRT6 through the AMPK–FoxO3a pathway. As Fig. 8A–D shows, oxyhemoglobin injury reduced the phosphorylation of AMPK and FoxO3a, which led to a decrease in the level of SIRT6. However, such deterioration was partially reversed by FGF21. To further clarify our understanding of this mechanism, we determined the effects of Compound C, an AMPK blocker, on the AMPK–FoxO3a pathway. Our results showed that the improvements in p-AMPK and p-FoxO3a were reversed by Compound C. Meaningfully, the expression of SIRT6 was also consistent with that of p-AMPK and p-FoxO3a after Compound C treatment. Therefore, our results reveal that FGF21 might increase the expression of SIRT6 through the AMPK–FoxO3a pathway. A schematic figure depicts such a mechanism (Fig. 8E).

## Discussion

As an endocrine factor mainly released by the liver, FGF21 has multiple beneficial abilities, such as controlling blood glucose, regulating energy expenditure, exerting anti-inflammatory effects, maintaining the function of mitochondria, and prolonging lifespan by acting in many different organs and tissues (Dolegowska et al. 2019; Fisher and Maratos-Flier 2016; Tezze et al. 2019; Geng et al. 2020; Kharitonenkov et al. 2005; Salminen et al. 2017; Kharitonenkov and Adams 2014). For example, a series of studies have clarified that FGF21 is capable of controlling plasma glucose in diabetic mice by improving their insulin sensitivity (Kang et al. 2020; Li et al. 2019; Morgeson et al. 2013). In addition, FGF21 has been claimed to have beneficial regulatory effects on lipid metabolism and thermogenesis, especially in mice undergoing serious stresses such as starvation, cold exposure and hepatic steatosis (Mai et al. 2009; Inagaki et al. 2008; Fisher et al. 2012; Zhang et al. 2015). Interestingly, in light of its ability to penetrate the BBB (Kharitonenkov and Adams 2014), recent studies have revealed that the systematic regulatory effects of FGF21 on metabolic homeostasis rely on the cooperation of the nervous system (Giralt et al. 2015; Owen et al. 2015). After FGF21 injection into cerebrospinal fluid, an increase in energy expenditure and insulin sensitivity can be observed (Xu et al. 2009a). In addition, recent studies have demonstrated that the expression of $\beta$-klotho in the suprachiasmatic nucleus and paraventricular nucleus of the hypothalamus is essential for the FGF21 signaling cascade (Bookout et al. 2013; Liang et al. 2014). These studies have demonstrated crosstalk between FGF21 and the nervous system. In turn, during some harmful events related to the nervous system, such as TBI, I/R injury, cognitive impairment and neurodegenerative diseases (Chen et al. 2018; Wang et al. 2018b; Kang et al. 2020; Jiang et al. 2019; Wang et al. 2020), FGF21 can also exert certain protective effects. Nevertheless, whether FGF21 has certain protective effects in ICH injury has yet to be elucidated.

The current study revealed four main findings; (a) for ICH patients, the serum level of FGF21 is positively correlated with the $\Delta$GCS%; (b) FGF21 can alleviate neural disorders by protecting the BBB after ICH in mice; (c) FGF21 is capable of protecting HCMECs by alleviating mitochondrial damage through the SIRT6 pathway in vitro; and (d) the SIRT6 pathway is involved in the protective effects of FGF21 in vivo, as confirmed via comparison of SIRT6^flox/flox^ and eSIRT6^−/−^ mice. Hereafter, we will discuss these 4 findings in detail.

The brain experiences a rapid rise in intracranial pressure during the onset of ICH. With this extremely altered pressure, many harmful cytokines consistently leak through the damaged BBB, which may trigger secondary damage, such as enlargement of the hematoma or brain edema (Lok et al. 2007). Therefore, maintaining the normal function of the BBB is an ideal strategy for breaking such a vicious cycle. Yinghua Jiang et al. declared that FGF21 could mitigate BBB damage after ischemic focal stroke through PPAR$\gamma$ activation in vivo (Jiang et al. 2020). The permeability of HCMECs was partially restored in vitro after FGF21 administration. Other authors, such as Mamtilahun et al., have also confirmed the therapeutic potential of FGF21 for the BBB by injecting healthy mouse plasma into mice with ischemic stroke (Mamtilahun et al. 2021). Upon damage caused by TBI, FGF21 continues to help protect the BBB (Chen et al. 2018). In the present study, after ICH, 77 ICH patients’ serum FGF21 levels and $\Delta$GCS% scores were measured and analyzed. We identified a positive correlation between the serum level of FGF21 and the $\Delta$GCS% in ICH patients, which indicates that FGF21 may be a candidate predictive marker for the prognosis of coma severity after ICH. In mice, immediate administration of FGF21 after ICH for 7 days dramatically improved the performance of mice in neurofunction tests. Moreover, by means of EB staining, we confirmed the protective effect of FGF21 on the BBB in vivo. Moreover, the levels of ZO-1 and occludin in proteins extracted from fresh samples were partially restored. These results not only are in line with previous studies but also further demonstrate that FGF21 can exert protective effects after ICH.

To explore the underlying mechanisms of such beneficial effects on the BBB after ICH, we reviewed a series of studies regarding FGF21. According to these studies, the specificity of FGF21 in different tissues depends on the presence of a cofactor,$\beta$-Klotho protein (Owen et al. 2015; Zhang et al. 2006). The formation of FGF21/FGFR1/$\beta$-Klotho compounds is a vital initial process for exerting normal biological function (Fisher et al. 2011; Xu et al. 2009b; Adams et al. 2012). However, after the initial procedure, subsequent downstream pathways related to different functions remain under debate. Importantly, in the present study, obvious improvements in mitochondrial morphology and function were observed after FGF21 treatment, and it is well known that damaged mitochondrial cristae contribute to the overwhelming leakage of reactive oxygen species, which ultimately leads to cell death (Murphy 2009). Considering such an intriguing finding, it is rational to focus on certain pathways related to mitochondrial homeostasis.

Sirtuins consist of 7 NAD^+^-dependent deacylase enzymes (SIRT1–SIRT7) that are located in different cellular compartments. Among them, SIRT6 is located mainly in the nucleus and has attracted increasing attention for its multiple beneficial biological functions, such as regulation of DNA double-strand break repair, anti-inflammatory effects, and functions in glucose homeostasis (Singh et al. 2018; Gertler and Cohen 2013). Recent studies have demonstrated that SIRT6 plays an important role in regulating mitochondrial quality and antioxidant signaling (Singh et al. 2018). According to Fan et al. (2019), SIRT6 is involved in mitigating the damage to mitochondria in podocytes induced by high glucose in vitro and in vivo through the AMPK pathway. Additionally, in cardiomyocytes, SIRT6 plays an important role in maintaining the normal morphology and function of mitochondria in vitro and in vivo (Yu et al. 2021; Cheng et al. 2016). Furthermore, through the genetic engineering of SIRT6^MCKO^ mice, Xiaona Cui et al. (Cheng et al. 2016) demonstrated that SIRT6 is implicated in the regulation of mitochondrial homeostasis in skeletal muscle through the AMPK pathway. These studies demonstrate that SIRT6 is involved in maintaining normal mitochondrial function and morphology in multiple organs. Moreover, SIRT6 has been confirmed to exert a series of protective effects on the nervous system in multiple diseases (Liberale et al. 2020; Zhang et al. 2017; Hu et al. 2015; Zuo et al. 2019). Nevertheless, the following question still needs to be answered: after treatment with FGF21, is SIRT6 involved in regulating the beneficial effects of FGF21 on mitochondria following ICH injury? Therefore, in the present study, a series of experiments was performed to verify the involvement of the SIRT6 pathway in vitro and in vivo. First, we observed a decrease in the immunofluorescence signal of SIRT6 in fresh slices after ICH. This decrease was partially reversed by FGF21 administration in vivo. In vitro, the expression of SIRT6 in the oxyhemoglobin-treated group was also decreased compared to that in the control group, but in the FGF21-treated group, the level of SIRT6 was again partially restored, which was consistent with the in vivo result. Intriguingly, after the administration of FGF21, with the improvement in SIRT6, the mitochondrial dysfunction caused by oxyhemoglobin was partially reversed. These results imply that SIRT6 might be involved in the downstream signaling cascade of FGF21. To further confirm the involvement of SIRT6 in vitro, we utilized siSIRT6 to interfere with SIRT6 expression. After SIRT6 interference, we observed that FGF21 lost the ability to preserve normal mitochondrial morphology and function. Furthermore, in vitro, the restoration of HCMEC monolayer permeability by FGF21 also disappeared. These results imply that FGF21 exerts protective effects on HCMECs by maintaining normal mitochondrial function through the SIRT6 pathway in vitro. According to previous literature, SIRT6 is a novel direct transcriptional target of FoxO3a (Zhang et al. 2019; Dong et al. 2020). Through qPCR, we revealed that SIRT6 mRNA expression in ECs was partly decreased after siRNA-FoxO3a interference in vitro. Additionally, we further assessed the expression of the AMPK–FoxO3a pathway, which plays a vital role mediating energy homeostasis under energy stress (Zhong et al. 2023). Through alterations in the relative protein levels among the different groups, we revealed that FGF21 might regulate SIRT6 expression through the AMPK–FoxO3a pathway after injury.

Finally, through genetic engineering of eSIRT6^−/−^ mice, we were the first to explore the relationship between FGF21 and SIRT6 in vivo. In contrast to the observations in their SIRT6^flox/flox^ littermates, FGF21 administration failed to mitigate the neural disorder after ICH in eSIRT6^−/−^ mice. In addition, although both were administered FGF21, the leakage of Evans blue in eSIRT6^−/−^ mice was more severe than that in their SIRT6^flox/flox^ littermates after ICH.

There were some limitations to the present study. Although we evaluated the mechanisms observed in vitro with eSIRT6^−/−^ mice, there were some differences between the ICH models in vitro and in vivo. Further study needs to be performed to explore the detailed mechanisms. In addition, we employed $\beta$-actin as a loading control to detect differences among multiple proteins, which might have brought certain bias attributed to its alterations after ICH injury. We demonstrated that FGF21 increased the expression of SIRT6 after ICH to maintain the dynamic morphology of mitochondria in HCMECs; however, more precise regulatory mechanisms between FGF21 and SIRT6 still need to be further elucidated. In this study, FGF21 alleviated ICH injury by increasing the expression of SIRT6 after injury. Whether directly activating SIRT6 can also exert certain protective effects after ICH was not assessed in this study. According to Cheng et al. (2023), overexpression of SIRT6 can attenuate ICH injury by downregulating NF-kB, which will undoubtedly shed light for our next step in exploration.

## Conclusion

In conclusion, this study indicates that FGF21 plays a beneficial role in ICH injury by preserving BBB integrity. At the molecular level, FGF21 might maintain mitochondrial dynamic morphology and function through the SIRT6 pathway to alleviate oxidative stress in HCMECs. The results from the present study shed light on the potential therapeutic value of FGF21.

### Supplementary Information

**Additional file 1. **Schematic figures for protocols of our experiment. (A) To assess the clinical value of FGF21, of 77 ICH patients were enrolled. According to the ∆GCS%, patients were divided into 2 groups: ∆GCS% > 0 group and ∆GCS% < 0 group. Their blood samples were collected to determine the relationship between the serum level of FGF21 and the ∆GCS%. (B) WT mice were divided into 4 groups to determine the protective effects of FGF21 for neural system and BBB in vivo. (C) HCMECs were cultivated and assigned into different groups to further explore the potential mechanism of FGF21 in vitro. (D) Gene engineered mice (SIRT6^flox/flox^ mice & eSIRT6^−/−^ mice) were constructed and compared among different treatments to verify relative mechanism.

**Additional file 2. **(A). qPCR for SIRT6 mRNA between si-NC and siSIRT6 groups in vitro. n = 6 per group. (B). qPCR for SIRT6 mRNA between SIRT6^flolx/flox^ and eSIRT6^−/−^ mice in vivo. n = 6 per group. (C). qPCR for SIRT6 mRNA between si-NC and siFoxO3a groups in vitro. n = 6 per group. ^***^P < 0.001 indicates si-NC vs siSIRT6 group by student’s t test (A), ^***^P < 0.001 indicates SIRT6^flox/flox^ mice vs eSIRT6^−/−^ mice by student’s t test (B), ^***^P < 0.001 indicates si-NC vs siFoxO3a group by student’s t test (C), Values are presented as mean  ± SD.

**Additional file 3. **Modified Neurological Severity Score (MNSS).

## Data Availability

The datasets used and/or analyzed during the current study are available from the corresponding author on reasonable request.

## Abbreviations

ICH: Intracranial hemorrhage
BBB: Blood–brain barrier
FGF21: Fibroblast growth factor 21
SIRT6: Deacylase sirtuin 6
CNS: Central nervous system
ECs: Endothelial cells
HCMECs: Human brain microvascular endothelial cells
ROS: Reactive oxygen species
TJ-ps: Tight junction proteins
AJ-ps: Adherens junction proteins
siSIRT6: Small interfering siRNA for SIRT6
si-NC: Negative control small interfering RNA
eSIRT6^−^^/^^−^ mice: Endothelial-specific homozygous-SIRT6-knockout mice
