# Supplementary figures and images for: FGF21 alleviates endothelial mitochondrial damage and prevents BBB from disruption after intracranial hemorrhage through a mechanism involving SIRT6
Source: Mol Med. 2023 Dec 4;29:165. doi: 10.1186/s10020-023-00755-x (PMC10696847; doi:10.1186/s10020-023-00755-x)

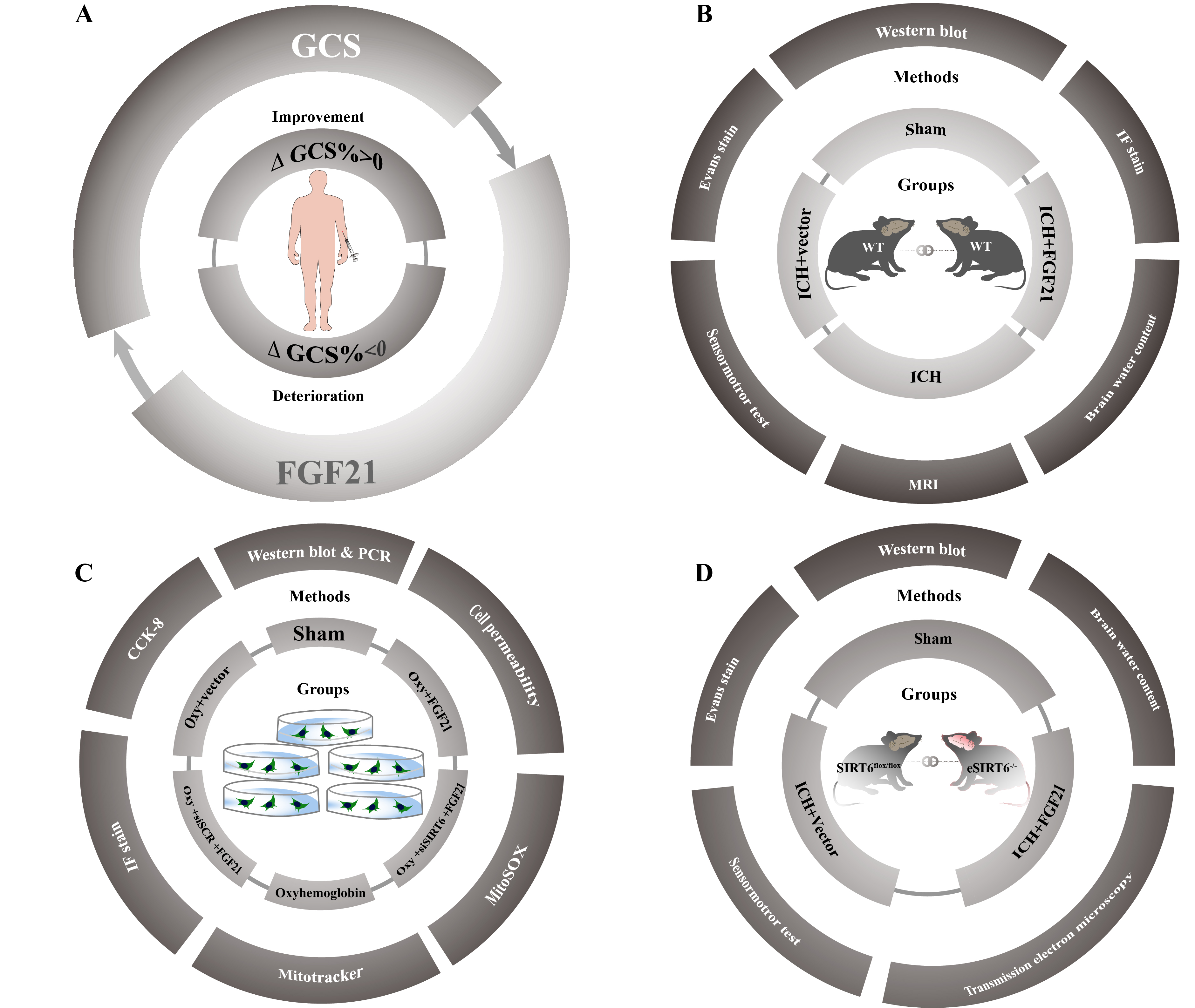

Supplement: Supplementary file 1 — Additional file 1. Schematic figures for protocols of our experiment. (A) To assess the clinical value of FGF21, of 77 ICH patients were enrolled. According to the ∆GCS%, patients were divided into 2 groups: ∆GCS% > 0 group and ∆GCS% < 0 group. Their blood samples were collected to determine the relationship between the serum level of FGF21 and the ∆GCS%. (B) WT mice were divided into 4 groups to determine the protective effects of FGF21 for neural system and BBB in vivo. (C) HCMECs were cultivated and assigned into different groups to further explore the potential mechanism of FGF21 in vitro. (D) Gene engineered mice (SIRT6flox/flox mice & eSIRT6−/− mice) were constructed and compared among different treatments to verify relative mechanism. [file 10020_2023_755_MOESM1_ESM.jpg]

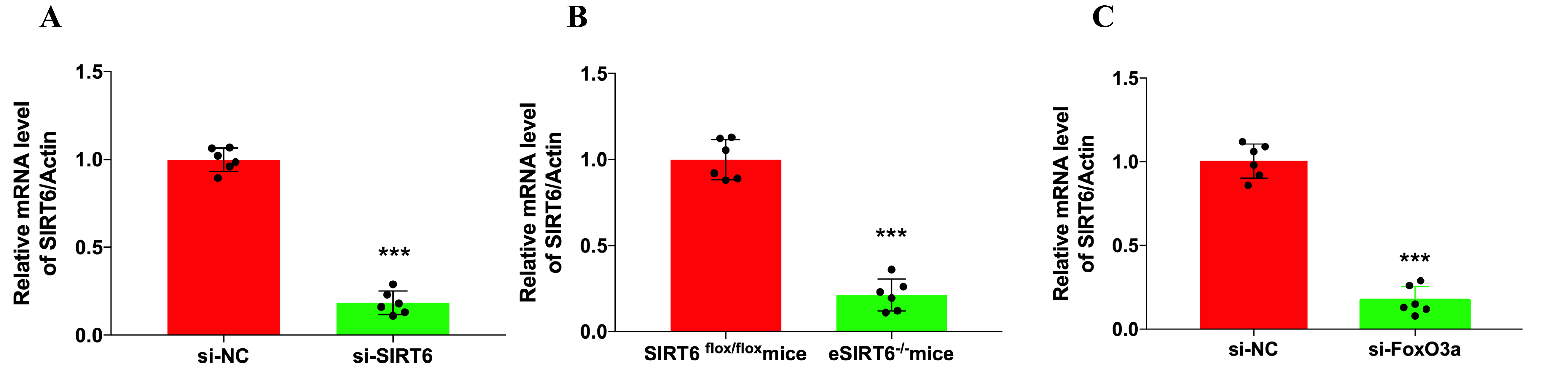

Supplement: Supplementary file 2 — Additional file 2. (A). qPCR for SIRT6 mRNA between si-NC and siSIRT6 groups in vitro. n = 6 per group. (B). qPCR for SIRT6 mRNA between SIRT6flolx/flox and eSIRT6−/− mice in vivo. n = 6 per group. (C). qPCR for SIRT6 mRNA between si-NC and siFoxO3a groups in vitro. n = 6 per group. ***P < 0.001 indicates si-NC vs siSIRT6 group by student’s t test (A), ***P < 0.001 indicates SIRT6flox/flox mice vs eSIRT6−/− mice by student’s t test (B), ***P < 0.001 indicates si-NC vs siFoxO3a group by student’s t test (C), Values are presented as mean ± SD. [file 10020_2023_755_MOESM2_ESM.jpg]

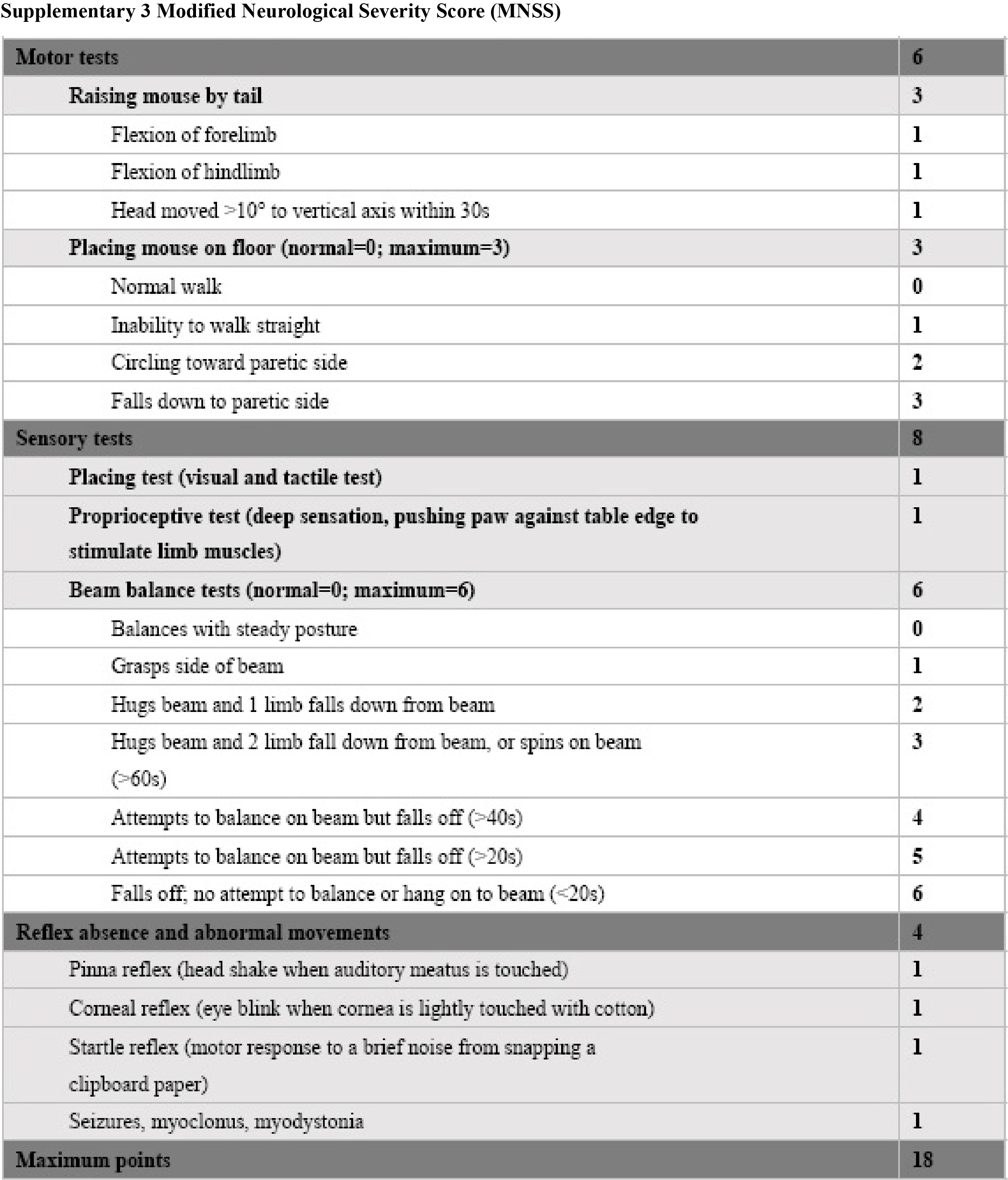

Supplement: Supplementary file 3 — Additional file 3. Modified Neurological Severity Score (MNSS). [file 10020_2023_755_MOESM3_ESM.jpg]
